# End-stage renal disease preceded by rapid declines in kidney function: a case series

**DOI:** 10.1186/1471-2369-12-5

**Published:** 2011-02-01

**Authors:** Peter Lee, Kirsten Johansen, Chi-yuan Hsu

**Affiliations:** 1 Division of Nephrology, University of California, San Francisco, San Francisco, CA, USA

## Abstract

**Background:**

Few studies have defined alternate pathways by which chronic kidney disease (CKD) patients transition into end-stage renal disease (ESRD).

**Methods:**

We studied all consecutive patients initiated on maintenance hemodialysis or peritoneal dialysis over several years at two dialysis units in Northern California. Rapid decline in kidney function was considered to have occurred if a patient was documented to have estimated GFR > 30 ml/min/1.73 m^2 ^within three months prior to the initiation of chronic dialysis.

**Results:**

We found that 8 out of 105 incident chronic dialysis patients one dialysis unit (7.6%; 95% confidence interval 3.4-14.5%) and 9 out of 71 incident patients at another (12.7%, 95% CI 6.0%-22.7%) suffered rapid decline in kidney function that was the immediate precipitant for the need for permanent renal replacement therapy. All these patients started hemodialysis and all relied on catheters for vascular access. Documentation submitted to United States Renal Data System did not fully reflect the health status of these patients during their "pre-ESRD" period.

**Conclusions:**

A sizeable minority of ESRD cases are preceded by rapid declines in kidney function. The importance of these periods of rapid decline may have been under-appreciated in prior studies of the natural history of CKD and ESRD.

## Background

Although there is a large body of epidemiology literature on patients with end-stage renal disease (ESRD)--and in the past decade, also on patients with chronic kidney disease (CKD)--relatively few studies have focused on the transition from CKD to ESRD. We hypothesize based on our clinical experience that in a sizable minority of incident ESRD patients develop ESRD after suffering period of rapid loss of glomerular filtration rate (GFR) which may or may not fit the current definition of acute kidney injury (abrupt rise in serum creatinine of ≥0.3 mg/dl or ≥50% within 48 hours)[1]. This has a number of clinical and policy implications which we believe deserve consideration.

The purpose of this study is to quantify the frequency with which chronic dialysis was precipitated by periods of rapid decline in kidney function at two dialysis units in Northern California, USA.

## Methods

We conducted a retrospective chart review study at two outpatient chronic dialysis units in San Francisco, California, USA. The study population consisted of consecutive patients initiated on chronic maintenance dialysis between October 2005 and December 2007 at California Pacific Medical Center (CPMC) Pacific Campus outpatient dialysis unit as well as consecutive patients initiated between January 2005 to December 2009 at San Francisco Veterans Affairs Medical Center (SFVAMC). CPMC is a private, not-for-profit medical center in San Francisco, California. SFVAMC is one of the three major teaching hospitals affiliated with University of California, San Francisco. Patients initiated on either chronic hemodialysis or peritoneal dialysis were included.

Medical records were reviewed to determine the fraction of patients whose ESRD was immediately preceded by periods of rapid decline in kidney function. Rapid decline in kidney function was considered to have occurred if a patient was documented to have estimated GFR > 30 ml/min/1.73 m^2 ^within three months prior to the initiation of chronic dialysis. GFR was estimated using the 4-variable Modification of Diet in Renal Disease (MDRD) equation [2,3]. We chose the MDRD equation because it was developed in a CKD population with a range of GFR similar to the range of GFR prior to ESRD we expected to examine [2].

We reviewed the preceding renal function trajectory (for up to 2 years prior to ESRD) for those patients meeting criteria for rapid decline in kidney function. All serum creatinine values available from the CPMC and the SFVAMC electronic laboratory databases were abstracted. In addition, for the patients who suffered rapid decline in kidney function, individual medical charts were reviewed in depth by an attending nephrologist (KJ or CYH) to determine the cause of the rapid decline. Hospital admission, discharge, and progress notes, as well as consultation reports, laboratory tests and imaging studies were reviewed as needed. Particular attention was paid to the etiology of rapid decline as determined by the attending nephrologist who cared for the patient.

For the CPMC patients, we also compared information abstracted from the medical record with that documented on the Medical Evidence (CMS 2728) Form, which is required of all new maintenance dialysis patients in the USA. This information was not accessible to researchers at the SFVAMC.

The study was approved by the local Institutional Review Boards. Because of the low risk nature of the study, individual patient consent was waived.

## Results

**Figure 1:**
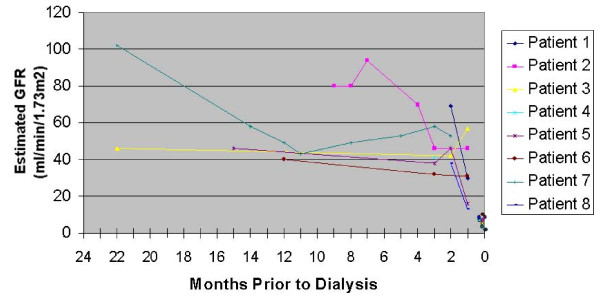
**Renal trajectory for the 8 ESRD cases immediately preceded by ARF from California Pacific Medical Center**.

Between October 2005 and December 2007, there were 105 incident chronic dialysis patients at CPMC. We found that among them, 8 (7.6%; 95% confidence interval 3.4-14.5%) suffered rapid decline in kidney function that was the immediate precipitant for the need for permanent renal replacement therapy. The renal trajectories of these 8 patients prior to ESRD are shown in Figure 1. Mean baseline estimated GFR prior to the rapid decline (defined as the highest GFR within 3 months) was 48.3 ± 12.3 ml/min/1.73 m^2 ^for these 8 patients.

**Figure 2:**
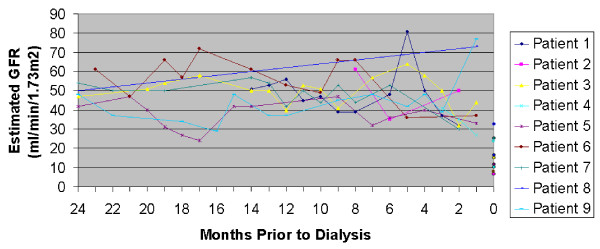
**Renal trajectory for the 9 ESRD cases immediately preceded by ARF from San Francisco Veterans Affairs Medical Center**.

Between January 2005 to December 2009, there were 71 incident chronic dialysis patients at SFVAMC. We found that among them, 9 (12.7%, 95% confidence interval 6.0%-22.7%) suffered rapid decline in kidney function that was the immediate precipitant for the need for permanent renal replacement therapy. The renal trajectories of these 9 patients prior to ESRD are shown in Figure 2. (The patients who were initiated with relatively higher estimated GFR required dialysis for volume control [e.g. cardiorenal physiology].) Mean baseline estimated GFR prior to the rapid decline was 50.6 ± 16.1 ml/min/1.73 m^2 ^for these 9 patients.

Table 1 and 2 show the characteristics of the ESRD patients whose ESRD was immediately preceded by rapid decline in kidney function compared with those whose ESRD was not. Those who developed ESRD immediately preceded by rapid decline in kidney function were all treated with hemodialysis and all had to rely on catheters for access (Tables 1 and 2). In contrast, of the incident ESRD cases not precipitated by rapid decline in kidney function at CPMC, 5.2% patients started peritoneal dialysis and approximately a quarter had mature arteriovenous fistulas. At SFVAMC, 19.3% of the incident ESRD patients who did not experience prior rapid decline in kidney function had fistulas, 3.2% of patients had grafts.

**Table 1:** California Pacific Medical Center patient characteristics

	ESRD precipitated by ARF (N = 8)	ESRD not precipitated by ARF (N = 97)
Age at dialysis initiation (yrs)	69.9 ± 19.2	66.9 ± 13.5

Male	4 (50%)	49 (50.5%)

Race/Ethnicity, N (%)		
White	3 (37.5%)	44 (45.3%)
Asian	5 (62.5%)	30 (30.9%)
Black	0	21 (21.6%)
Hispanic	0	2 (2.1%)

Co-morbid conditions, N (%)		
Hypertension	6 (75%)	85 (87.6%)
Diabetes mellitus	4 (50%)	41 (42.2%)
Congestive heart failure	1 (12.5%)	20 (20.6%)

Initial renal replacement therapy modality, N (%)		
Hemodialysis	8 (100%)	92 (94.8%)
Peritoneal dialysis (PD)	0	5 (5.2%)*

Access for dialysis, N (%)		
Hemodialysis catheter	8 (100%)	69 (71.1%)¶
Hemodialysis fistula	0	22 (22.7%)
Hemodialysis graft	0	1 (1.0%)
Peritoneal dialysis catheter	0	5 (5.2%)

* p = 0.67 (Fisher's exact test)

¶ p = 0.33 (Fisher's exact test)

**Table 2:** San Francisco Veterans Affairs Medical Center patient characteristics

	ESRD precipitated by ARF (N = 9)	ESRD not precipitated by ARF (N = 62)
Age at dialysis initiation (yrs)	69.7 ± 10.5	67.3 ± 12.2

Male	9 (100%)	58 (93.5%)

Race/Ethnicity, N (%)		
White	2 (22.2%)	27 (43.5%)
Asian	1 (11.1%)	13 (20.9%)
Black	6 (66.7%)	22 (35.5%)
Hispanic	0	0

Co-morbid conditions, N (%)		
Hypertension	9 (100%)	56 (90.3%)
Diabetes mellitus	5 (55.6%)	35 (56.5%)
Congestive heart failure	1 (11.1%)	24 (38.7%)

Initial renal replacement therapy modality, N (%)		
Hemodialysis	9 (100%)	62 (100%)
Peritoneal dialysis (PD)	0	0

Access for dialysis, N (%)		
Hemodialysis catheter	9 (100%)	48 (77.4%)§
Hemodialysis fistula	0	12 (19.3%)
Hemodialysis graft	0	2 (3.2%)
Peritoneal dialysis catheter	0	0

§ p = 0.50 (Fisher's exact test)

The etiologies of the rapid decline in kidney function leading to ESRD--as determined by retrospective chart review by the study investigators--are shown in Tables 3 and 4. Among the 8 patients at CPMC whose ESRD was precipitated by rapid decline in kidney function, the etiology of ESRD listed in the Medical Evidence Form suggested the acute component in 6 (such as cholesterol emboli, hemolytic-uremic syndrome, glomerulonephritis and acute tubular necrosis). In the remaining 2 patients, it was not possible to discern that there was an acute component to their disease trajectory (since only diabetes mellitus was listed)(Table 3).

**Table 3:** Cause of ESRD as determined by chart review (vs. as reported in the Medical Evidence Form) among California Pacific Medical Center patients

Patient	Cause as determined by chart review	Documented cause reported on Medical Evidence Form
1	Cholesterol emboli	Cholesterol emboli

2	Hemolytic uremic syndrome	Hemolytic uremic syndrome

3	Acute tubular necrosis	Type 2 diabetes mellitus

4	Polyarteritis	Polyarteritis

5	Cholesterol emboli	Type 2 diabetes mellitus

6	Cyclosporine toxicity	Complications of transplanted heart

7	Gemcitabine toxicity	Tubular necrosis

8	Cholesterol emboli	Cholesterol emboli

**Table 4:** Cause of ESRD as determined by chart review among San Francisco Veterans Affairs Medical Center patients

Patient	Cause as determined by chart review
1	Acute tubular necrosis

2	Acute tubular necrosis

3	Acute tubular necrosis

4	Unable to determine

5	Acute tubular necrosis

6	Acute tubular necrosis

7	Acute tubular necrosis

8	Acute tubular necrosis

9	Acute tubular necrosis

Finally, based on data from CPMC, we found that the documented hemoglobin and albumin levels in the Medical Evidence Form reflected values observed at the start of acute dialysis and not values when the patients were in their usual "baseline" state of health prior to rapid decline in kidney function (Table 5).

**Table 5:** Comparison of laboratory values before and during acute renal failure among California Pacific Medical Center patients

Patient		3 Months Prior to Dialysis	Immediately preceding start of dialysis	Recorded on 2728 Form
1	Creatinine (mg/dl)	1.1	15.1	15.1
	
	Albumin (g/dl)	3.8	2.7	2.7
	
	Hemoglobin (g/dl)	13.8	11.9	11.9


2	Creatinine (mg/dl)	1.3	5.9	5.9
	
	Albumin (g/dl)	3.8	3.0	N/A
	
	Hemoglobin (g/dl)	9.9	10.3	10.3


3	Creatinine (mg/dl)	1.0	6.0	6.0
	
	Albumin (g/dl)	3.8	3.6	3.2
	
	Hemoglobin (g/dl)	11.9	10.0	10.0


4	Creatinine (mg/dl)	1.7	7.9	7.9
	
	Albumin (g/dl)	N/A	2.1	2.0
	
	Hemoglobin (g/dl)	N/A	8.3	8.3


5	Creatinine (mg/dl)	1.2	5.1	5.5
	
	Albumin (g/dl)	3.5	3.2	3.3
	
	Hemoglobin (g/dl)	11.8	13.6	13.3


6	Creatinine (mg/dl)	2.6	6.5	5.4
	
	Albumin (g/dl)	4.2	4.2	4.0
	
	Hemoglobin (g/dl)	12.6	10.1	10.2


7	Creatinine (mg/dl)	1.3	11.2	11.0
	
	Albumin (g/dl)	4.6	2.5	2.5
	
	Hemoglobin (g/dl)	12.6	10.3	11.1


8	Creatinine (mg/dl)	1.4	4.7	4.7
	
	Albumin (g/dl)	3.4	3.1	3.1
	
	Hemoglobin (g/dl)	11.1	10.2	10.2

N/A = not available

To convert creatinine to μmol/L, multiple by 88.4

To convert albumin to g/L, multiple by 10

To convert hemoglobin to g/L, multiple by 10

## Discussion and Conclusion

In this study of consecutive patients initiated on dialysis at two San Francisco outpatient dialysis units, we found there was a period of rapid decline in kidney function prior to the need for permanent dialysis for 7.6% of patients at one unit and 12.7% of patients at another. All patients who started chronic dialysis precipitated by rapid decline in kidney function started hemodialysis and all relied on catheters as access. For these patients, the "primary etiology" of ESRD recorded on the ESRD Medical Evidence (CMS 2728) Form, such as diabetes mellitus, sometimes did not capture the critical role of this acute episode in precipitating the need for ESRD therapy. Furthermore, the creatinine and hemoglobin values documented in the Medical Evidence form did not fully capture the clinical status of these patients during their "pre-ESRD" period.

Few prior studies have taken our study approach and examined relatively abrupt transitions to ESRD at the individual-patient level [4,5]. Although our results are based on a relatively small number of patients from two hospital-affiliated dialysis units, we believe that our results have face validity and are consistent with the clinical experience of practicing nephrologists. Our results are also consistent with that reported by Bhandari and Turney, who found that from 1984 to 1995, survivors of "acute renal failure" who did not regain renal function comprised 18.4% of all new patients taken into their long-term dialysis program in England [4]. They are also consistent with the high rates of non-recovery of renal function following dialysis requiring acute kidney injury which have been reported among patients ware critically ill [6] or who have pre-existing chronic kidney disease [7].

We believe that these findings should stimulate thinking along several lines. First, periods of rapid loss of renal function appear to be a prominent feature of the natural history of kidney disease in a sizable minority of patients who develop ESRD. For example, Patient 1 at CPMC had a decline in estimated GFR from 69 ml/min/1.73 m^2 ^to ESRD within a two-month period due to cholesterol emboli. More research is needed to investigate whether other CKD patients (e.g., those who did not develop ESRD in the short run) also experience these periods of rapid decline in renal function as part of the natural history of their disease.

Second, patients who start chronic dialysis as a result of rapid loss of kidney function over a short period of time will naturally have little, if any, opportunity to be educated about dialysis modality choices or undergo adequate preparation for dialysis such as pre-emptive creation of an arteriovenous fistula. Hence, it is not surprising that we observed that 100% of all ESRD cases preceded by rapid decline in kidney function were initiated on hemodialysis via a catheter. These considerations suggest that national policy targets such as that set by "Fistula First" in the USA must take into account the proportion of new dialysis patients whose ESRD was precipitated by rapid decline in kidney function. For example, one of the original clinical performance measures in the Center for Medicare & Medicaid Services ESRD US Clinical Performance Measures project was: "A primary arterial venous fistula should be the access for at least 50% of all new patients initiating hemodialysis [8]." We would suggest that the proportion of patients who started dialysis immediately precipitated by these rapid declines in kidney function should be removed from the numerator (and denominator) of such targets. In other words, the fact that some patients suffer rapid loss of kidney function and transition abruptly from mild-to-moderate CKD to dialysis-dependency must be taken into consideration when establishing appropriate clinical performance measures for incident dialysis patients. These patients will also likely not be evaluated for pre-emptive kidney transplantation.

Third, results of this study should inform our interpretation of the data in the US Renal Data System registry. The "primary etiology" of ESRD listed on the ESRD Medical Evidence (CMS 2728) Form may not convey the critical role of these episodes of rapid decline in kidney function in precipitating the need for ESRD therapy--e.g., in the example of Patient 3 at CPMC when there was severe acute tubular necrosis superimposed upon CKD due to diabetes mellitus. The traditional approach of ascribing only one single cause for each ESRD case in the American and other national registries does not capture the complexity of clinical reality. In addition, because of the existing data collection process, current documentation in the US Renal Data System may not accurately reflect the "pre-dialysis" health status in a number of patients. The hemoglobin and albumin at the start of dialysis as documented in the Medical Evidence form has been used in prior studies to assess quality of CKD care in the USA and to infer decisions that are made by patients and physicians regarding timing of initiation of dialysis [9,10]. These analyses assume that all dialysis patients had a slowly progressive CKD course and that there were opportunities available to treat the low hemoglobin level or to initiate dialysis before hypoalbuminemia developed. This assumption naturally does not hold in the cases where there are superimposed episodes of rapid decline in kidney function.

The strengths of this study include its emphasis on a hitherto underappreciated aspect of the natural history of transition from CKD to ESRD. We studied this issue in two distinct health care settings which serve different patient populations and this strengthened the external validity of our conclusions. The clinical and public health importance of highlighting this "cause" of ESRD--affecting perhaps 5-10% of incident ESRD patients--should be put into context of other ESRD etiologies. By comparison, according to US Renal Data System, 3% of ESRD are due to "cystic/heriditary/congential diseases" and 2% to "secondary glomerulonephritis" (such as lupus nephritis) [11].

Limitations of this study relate to its small sample size which is partly due to the labor-intensive process of conducting chart review. However, the level of granularity achieved (down to exact day of initiation of acute dialysis which then transitioned into maintenance dialysis) may not be reliably replicated in administrative databases in which the exact start of date of dialysis may be documented with a margin of error of up to a few months. The small sample size limited our ability to conduct meaningful analyses regarding predictors of rapid decline in kidney function or whether the post-ESRD outcomes differed among patients who did and did not suffer rapid decline in kidney function. Our results are based on a two hospital affiliated dialysis units in Northern California and so may not be generalizable to other setting. The mean age and proportion of male patients in our study sample were higher than the general U.S. incident ESRD population. However, they have face validity and are consistent with the clinical experience of practicing nephrologists. Owing to the limited sample sizes, numerous comparisons (Tables 1 and 2) did not achieve statistical significance, although it seems unlikely that differences observed were merely chance findings (e.g., the pre-test probability is high that patients who develop acute kidney injury with non-recovery of renal function would not have mature dialysis fistulas in place). We were not able to access information documented on the Medical Evidence (CMS 2728) Form for patients at one of the two dialysis units. We had limited information about the setting in which the rapid declines in kidney function occurred and about the care provided to these patients (e.g. timing of nephrology involvement). Our definition of "rapid decline in kidney function" was arbitrary. We did not use consensus definitions for acute kidney injury that were developed more for hospitalized patients (especially critically ill inpatients) [1] as we wanted to capture a broader spectrum of rapid decline in renal function. But we believe that our estimation of the magnitude of the problem is conservative since we required documentation within three months of ESRD of a relatively high prior estimated GFR value of 30 ml/min/1.73 m^2^. Thus, a patient who has had stable GFR at 25 ml/min/1.73 m^2 ^for several years before developing ESRD abruptly as a result of severe acute tubular necrosis during a hospitalization would not have been counted; neither would a patient who presented with rapidly progressive glomerulonephritis without known prior serum creatinine measurements in the hospital systems.

In summary, it appears that numerous cases of ESRD are preceded by rapid declines in kidney function. The importance of these periods of rapid decline in kidney function has been under-appreciated in prior studies of the natural history of CKD and ESRD.

## Competing interests

The authors declare that they have no competing interests.

## Authors' contributions

CYH designed the study. PL acquired the data. PL, KJ and CYN analyzed and interpreted the data. CYH performed the statistical analysis. PL and CYH drafted the manuscript. KJ revised the manuscript for important intellectual content. PL, KJ, CYH read and approved the final manuscript.

## Pre-publication history

The pre-publication history for this paper can be accessed here:

http://www.biomedcentral.com/1471-2369/12/5/prepub
